# Malocclusion of Molar Teeth Is Associated with Activities of Daily Living Loss and Delirium in Elderly Critically Ill Older Patients

**DOI:** 10.3390/jcm10102157

**Published:** 2021-05-17

**Authors:** Yoshihisa Fujinami, Toru Hifumi, Yuko Ono, Masafumi Saito, Tomoya Okazaki, Natsuyo Shinohara, Kyoko Akiyama, Misa Kunikata, Shigeaki Inoue, Joji Kotani, Yasuhiro Kuroda

**Affiliations:** 1 Department of Disaster and Emergency and Critical Care Medicine, Kobe University Graduate School of Medicine, Kobe 650-0017, Japan; greatyoppie@yahoo.co.jp (Y.F.); windmill@people.kobe-u.ac.jp (Y.O.); masa9804chicco@gmail.com (M.S.); kotanijo0412@gmail.com (J.K.); 2 Department of Disaster and Emergency and Critical Care Medicine, Kagawa University Graduate School of Medicine, Kagawa 761-0701, Japan; hifumitoru@gmail.com (T.H.); tomoyaokazaki4028@gmail.com (T.O.); natsuyot@med.kagawa-u.ac.jp (N.S.); kyokoakiyama@med.kagawa-u.ac.jp (K.A.); misakunikata@med.kagawa-u.ac.jp (M.K.); kuroday@med.kagawa-u.ac.jp (Y.K.)

**Keywords:** poor oral health, frailty, ICU prognosis

## Abstract

A single-center retrospective cohort study examined the association between molar malocclusion status at ICU admission and loss of activities of daily living (ADL) at hospital discharge among acutely ill patients. Patients were assigned to the bilateral occlusion group or malocclusion group (*N* = 227 and 93, respectively). The following data were collected from electronic medical records: age, sex, Clinical Frailty Scale (CFS) on admission, Acute Physiology and Chronic Health Evaluation (APACHE) Ⅱ score, confirmed diagnosis (neurological disorders or others), CFS at hospital discharge, and occlusion condition. Patients who were frail at admission (CFS > 5) were excluded from analysis, and ADL loss was defined as CFS > 5 at hospital discharge. Multivariate analysis showed malocclusion was independently associated with ADL loss [OR, 2.03; 95% CI, 1.13–3.64; *p* = 0.02]. For those aged 65 and older, malocclusion was significantly associated with both ADL loss [OR, 3.25; 95% CI, 1.44–7.32; *p* < 0.01] and the incidence of delirium [OR, 2.61; 95% CI, 1.14–5.95; *p* = 0.02]. Malocclusion on ICU admission was associated with ADL loss in critically ill patients, and was associated with ADL loss and the incidence of delirium in the elderly. Poor oral health was a poor prognostic factor among critically ill patients.

## 1. Introduction

The increasing number of elderly patients admitted to ICUs is a major concern for healthcare management, as aging is a prognostic factor for mortality among ICU patients [1]. Indeed, a Canadian multicenter prospective cohort study revealed that 30% of ICU patients over the age of 80 remained in the unit for more than seven days, and 22% died while being there [2]. Therefore, clinicians have been searching for an intervening factor through which to improve the prognosis of aging patients in ICUs.

Because aging itself is inevitable, frailty has been recently regarded as important by clinicians. Frailty is the most challenging manifestations of the aging population [3]. It is defined as a clinically recognizable state of increased vulnerability resulting from aging-associated functional decline in multiple physiological systems [4]. A meta-analysis showed that the frailty of ICU patients was associated with higher hospital and long-term mortality, with lower discharges [5]. Notably, delirium—a neurobehavioral syndrome characterized by impaired cognition with nonspecific manifestations—is one of the reasons for the poor prognosis; it also affects long-term activities of daily living (ADL) and cognitive impairment [6].

Several studies have reported an association between oral health and the pathogenesis of frailty [7]. Elderly people with frailty have significantly poorer oral function than prefrail and robust individuals. Moreover, the risk of frailty has been associated with lower occlusal force, masseter muscle thickness, and oral diadochokinetic rate [8]. Patients with poor oral health have more comorbidities such as cardiovascular diseases and strokes [9], chronic obstructive pulmonary disease [10], and type 2 diabetes [11]. Furthermore, they tend to experience malnutrition [12] and cognitive dysfunction [13]. Beyond this, poor oral health is associated with low socioeconomic status [14].

When accompanied by impaired malocclusion status with tooth loss and periodontal disease, poor oral health is associated with mortality [15,16,17]. However, the association between malocclusion status (a principal component of oral frailty) and ADL loss or delirium among acutely ill patients remains unclear. Thus, this study serves to determine the relevance of malocclusion on ADL loss and delirium development among acutely ill patients.

## 2. Materials and Methods

### 2.1. Study Design and Setting

This retrospective cohort study was based in an emergency medical center at Kagawa University Hospital, Japan. The study was conducted according to the guidelines of the Declaration of Helsinki, and the protocol and statistical analyses plan were approved by the review board at Kagawa University Hospital (IRB number: H30-173).

### 2.2. Participants and Data Sources

The study included all patients aged ≥18 years who were admitted to the facility’s emergency ICU between 1 November 2017 and 31 October 2018. Patients who had been discharged from the hospital within 48 h, and patients who received palliative care were excluded. Patients who were frail on admission [i.e., Clinical Frailty Scale (CFS) > 5] were also excluded from analysis. Finally, we also excluded those with missing data, using complete data sets alone.

The following data were collected from the hospital’s electronic medical records: age, gender, CFS on admission, Acute Physiology and Chronic Health Evaluation (APACHE) Ⅱ score, confirmed diagnosis (neurological disorders or others), CFS at hospital discharge, and occlusion condition.

### 2.3. Definitions

#### 2.3.1. Bilateral Occlusion and Malocclusion

Occlusion condition was assessed according to the number of teeth, chewing ability, articulatory oral motor skill, tongue pressure, as well as subjective difficulty when eating tough foods and swallowing in general. Our database captured the information about the condition of molar teeth occlusion but did not record other items.

Since occlusal contact area and occlusal force show similar results [18,19], occlusions of the premolars and the molars were assessed. Bilateral occlusion status was defined as having at least one set of the molars on the same side of the upper and lower jaw. Although a similar evaluation method has not been used in the past, we set the current definition of malocclusion based on the Eichner index [20], which evaluates malocclusion based on the number of occlusal supports provided by the molars and premolars. Based on this definition, subjects were divided into a bilateral occlusion group and a malocclusion group. Subsequently, we excluded from the bilateral occlusion group when (1) the tooth crown was lost, even if the root of the tooth was still present; (2) the mobility of the occlusal tooth was Grade 3, shaky in three dimensions; and (3) there was an existing traumatic tooth injury. The occlusion condition was assessed by X-ray images or the dentist examinations held once a week as an ICU round.

#### 2.3.2. ADL Loss

To evaluate ADL loss, this study employed the CFS [21] (Appendix A). Specifically, ADL loss was defined as patients with a CFS > 5 at the time of hospital discharge, that is, they were completely dependent outside the facility. A score of 6 was deemed moderately frail, while 7 = severely frail, 8 = extremely frail, and 9 = terminally ill. Other studies have used this definition of ADL loss [22]. We chose not to include the patients with a CFS > 5 at the time of admission because it is difficult to distinguish hospital-acquired ADL loss from underlying etiologies.

Patients who died in hospital were categorized as an ADL loss group. Notably, CFS was scored using nursing records of patient activity and interviews with patient families. The CFS was independently estimated by the author, two physicians (T.O. and N.S.), and two nurses (K.Y. and M.K.). The consistency was 88% with a maximum difference of two points and Cohen’s kappa coefficient was 0.82. These raters independently determined the CFS score of each patient, and a third adjudicated any remaining conflicts after the score reconciliation.

#### 2.3.3. Neurological Disorders

The classification of neurological disorders was applied if the subject had cerebral hemorrhage, subarachnoid hemorrhage (SAH), cerebral infarction, epilepsy, encephalitis, hepatic encephalopathy, brain sarcoidosis, post resuscitation encephalopathy, or traumatic brain injury (TBI). Notably, TBI included traumatic SAH, brain contusion, epidural hematoma, and subdural hematoma. This category did not include head injury devoid of brain injury.

#### 2.3.4. Delirium

We diagnosed delirium based on the confusion assessment method for the ICU (CAM-ICU) [23] up to 14 days after admission. During endotracheal intubation, sedation was managed with a goal of RASS −2 to −1. CAM-ICU was assessed for each shift, and delirium was considered present if observed once during the ICU stay for up to two weeks after admission. Patients who died in hospital were categorized as a Delirium group. The patients in a comatose state with Glasgow Coma Scale (GCS) < 9 from admission onward were excluded. Patients with severe dementia were already excluded by the “CFS > 5 on admission” criterion. Patients who died in hospital were excluded from analysis.

### 2.4. Exposure and Outcome Measurement

The primary exposure was malocclusion status. The primary outcome was ADL loss and the secondary outcome was the occurrence of delirium.

### 2.5. Statistical Analysis

Differences in the baseline clinical characteristics of the bilateral occlusion group and the malocclusion group were evaluated. The Mann-Whitney U test was used to compare differences in the continuous variables between the two groups. Where appropriate, either the Fisher exact test or chi-squared test were used to compare differences in categorical variables between the two groups. A multivariate logistic regression analysis was used to adjust for the potential confounders of age, gender, CFS on admission, APACHE II score, and neurological disorders on admission. This yielded an adjusted odds ratio (OR) for ADL loss and the occurrence of delirium after malocclusion as the primary exposure. A set of these variables was chosen a priori, based on previous reports [16,17,21,22,24] and biological plausibility. Although recent studies on ICU patients and frailty have often applied the CFS to those over 18 years of age [5] as the present study, the CFS is originally designed as a tool to assess frailty in patients over 65 years of age. Therefore, we conducted subgroup analysis for the patients 65 years or older. All statistical analyses were performed using JMP software version 13 (SAS Institute, Cary, NC, USA). A two-sided probability value of <0.05 was considered statistically significant for all analyses.

## 3. Results

**Figure 1:**
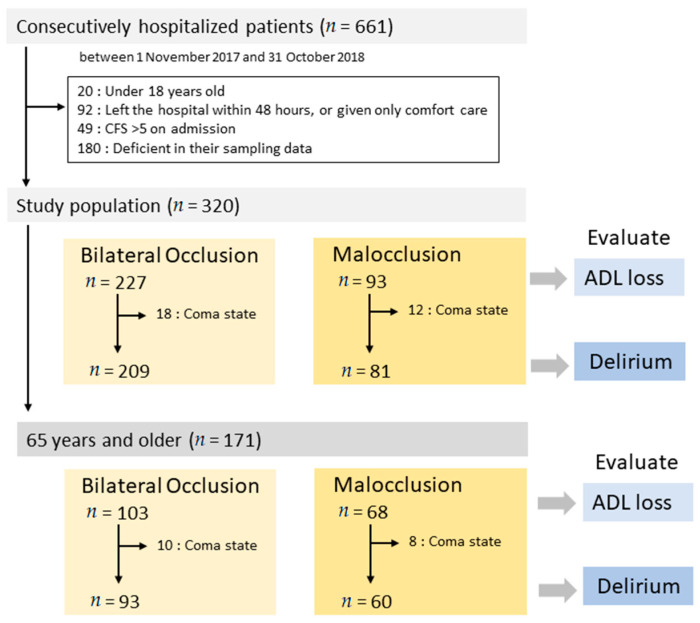
Study participants’ enrolment flow chart.

The sample included 661 patients, 320 of whom met the inclusion criteria (Figure 1). We used X-ray images to assess occlusal conditions in 228 patients, and 92 patients were assessed through dental examinations. The median age of patients was 69 and 56% were male. The median CFS on admission was 3, while that of the APACHE II score was 15. Forty percent of patients had neurological disorders. Sixteen patients (5.0%) died in hospital and 93 patients (29%) had ADL loss (Table 1). The bilateral occlusion group comprised 227 patients and the malocclusion group comprised 93. Age, CFS on admission, and APACHE II score were significantly higher for malocclusion patients than for bilateral occlusion patients (*p* < 0.01; Table 1).

In the analysis for those aged 65 and older, the median age of patients was 75 and 49% were male. The median CFS on admission was 3, while that of and APACHE II score was 16. Forty three percent of patients had neurological disorders. Ten patients (6.0%) died in hospital and 58 patients (34%) had ADL loss (Table 2). The bilateral occlusion group comprised 103 patients and the malocclusion group had 63. CFS on admission and APACHE II score were significantly higher for malocclusion patients than bilateral occlusion patients (*p* < 0.01; Table 2).

Regarding the primary endpoint, multivariate analysis showed that malocclusion was significantly associated with ADL loss [adjusted odds ratio (OR) 2.03; 95% confidence interval (CI), 1.13–3.64; *p* = 0.02] (Table 3). Notably, CFS on admission, APACHE II score, and neurological disorders were significantly associated with ADL loss, as well (Table 3).

In the multivariate analysis for those aged 65 and older, malocclusion was significantly associated with ADL loss [adjusted OR 3.25; 95% CI, 1.44–7.32; *p* = 0.02] (Table 4).

To allow for the investigation of patient delirium, those who were comatose or had severe dementia at admission were excluded. Thus, 290 patients were included: 209 patients in the bilateral occlusion group and 81 in the malocclusion group (Figure 1; Table 1).

Age, CFS on admission, APACHE II score, the percentage of neurological disorders, and the percentage of malocclusion were significantly higher among delirium patients than they were among non-delirium patients (Table 5). However, multivariate analysis revealed that malocclusion was not associated with the incidence of delirium among the acutely ill patients [adjusted OR, 1.33; 95% CI, 0.76–2.34; *p* = 0.32]. (Table 5).

In the multivariate analysis for those aged 65 and older, malocclusion was significantly associated with the incidence of delirium [adjusted OR, 2.61; 95% CI, 1.14–5.95; *p* = 0.02], (Table 6).

## 4. Discussion

This study found that malocclusion at admission was significantly associated with the development of ADL loss in ICU patients and the incidence of delirium in elderly people aged 65 years and older. This is the first study to demonstrate the impact that poor oral health has on ADL loss, leading to quality-of-life issues such as frailty and cognitive impairment in acutely ill patients. Notably, patients with malocclusion were older, frailer, and more critically ill at the time of admission.

Although ADL loss is well known to be associated with aging, frailty, and a high APACHE score [5], our study revealed that ADL loss was also associated with neurological disorders. There were significant differences between the bilateral occlusion and malocclusion groups regarding several of the baseline characteristics measured during admission, such as age, APACHE score, and CFS (Table 1 and Table 2). This study demonstrated that patients with malocclusion score significantly higher on these items than the bilateral occlusion group. Since they are well known as poor prognostic factors [5], such differences are often noteworthy problems for clinicians, especially during emergency medical treatment. Moreover, multivariate analysis revealed that malocclusion itself was an independently poor prognostic factor for acutely ill patients (Table 3). In this study, we excluded patients with CFS > 5, and they were mostly over 80 years old. Because moderately or severely frail patients at admission were excluded, the average age of the subjects was lower than that in other observational studies, and age was not associated with outcome. We have additionally analyzed the difference in ADL using the difference of CFS from at admission to at discharge. Furthermore, we conducted subgroup analysis by stratifying the level of CFS at admission to observe the difference of CFS in each level of ADL (Appendix B), because the situation is very different between CFS 2 to 4 and CFS 4 to 6 even though the CFS went up by 2. Even though the statistical power was decreased by stratification, we could observe the significant difference of CFS in CFS 3 group at admission (*p* = 0.02), and the trend of decrease in CFS difference of CFS 2 and 4 groups at admission (*p* = 0.09, 0.14 respectively). These data are consistent with our original data that malocclusion is associated with the development of ADL loss.

As previously noted, patients with poor oral health have more comorbidities [9], and poor oral health is associated with low socioeconomic status, as evidenced by education and income [14]. Notably, for some diseases, socioeconomic status is associated with prognosis [25,26]. Even in Japan, where health insurance is provided largely without exception, low socioeconomic status is correlated with major health disadvantages.

**Figure 2:**
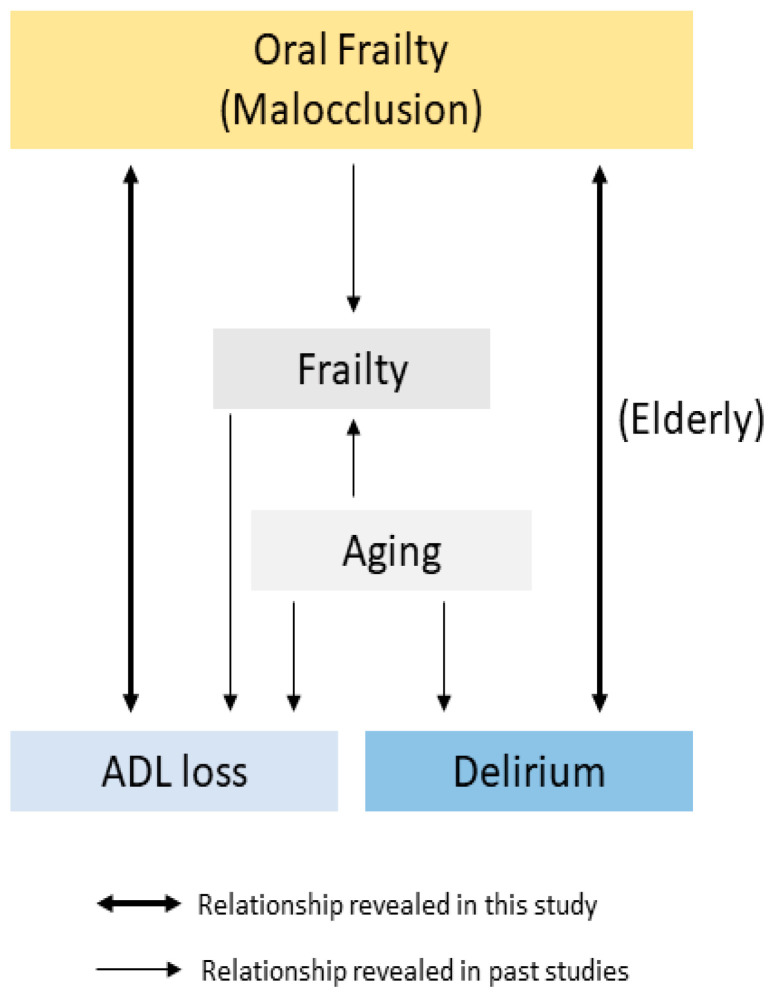
Correlation diagram of Aging, Frailty, and Oral Frailty.

Studies have shown that malocclusion is associated with frailty, and frailty is a poor prognostic factor [5,27]. Notably, our study showed that malocclusion was an independent factor of poor prognosis (Table 3; Figure 2). We provided information on quantifications of the interaction between the variables of the multivariate analyses (Appendix C), which demonstrated that interaction of malocclusion on age and severity was observed in both ADL loss and delirium. These results suggest that malocclusion has both direct and indirect effects on ADL loss. Although the relationship between malocclusion and ADL loss is still unclear, we speculate that malocclusion contributed to (1) recovery delay from a pathological condition, (2) secondary infection, (3) malnutrition, and (4) delirium. As the evidence for (1): There is a report that occlusal disharmonies cause immune system dysfunction and the malfunction of central catecholaminergic neurotransmission [28]. For (2): Many researchers have reported that poor oral health is associated with the occurrence of ventilator-associated pneumonia among ICU patients [24]. For (3): Fewer teeth are positively related to swallowing dysfunction, and poor oral health causes a decrease in saliva secretion; these mechanisms could contribute to malnutrition [29]. For (4): This study assessed the contribution to delirium as the secondary outcome because it is well known as a risk factor for poor prognosis in ICU patients [23].

Delirium in the ICU is often assessed with CAM-ICU [23] and predicted using the Early Prediction Model for Delirium in ICU Patients (E-PRE-DELITIC) model [30], which was proposed by Boogaard and colleagues. With reference to this model, this study adopted urgent admission, age, APACHE score, and neurological disorders for evaluation. Moreover, it was reported that frailty on admission was the risk factor for delirium in urgently admitted patients [31]; therefore, we adopted frailty as an item. In addition, we adopted malocclusion as an item to substantiate the hypothesis that malocclusion was associated not only indirectly but also directly.

Both oral health problems and cognitive impairment are relatively common among older adults and individuals with lower cognitive scores had a higher number of decayed and missing teeth and a higher proportion of periodontitis sites [32,33]. It has also been reported that cognitive impairment is associated with ICU delirium [34]. These findings corroborate the hypothesis that tooth loss may be a predictor or risk factor for ICU delirium [33]. In terms of delirium, ICU delirium is a factor that affects long-term ADL [35]. Moreover, it is a common complication among patients in the ICU [36], and it is associated with higher mortality, prolonged length of stay, and long-term cognitive impairment [37].

Several mechanisms may explain the association between malocclusion and delirium/cognitive impairment. First, the association may stem from chronic inflammation due to periodontal disease, which is a major cause of tooth loss in later life. Notably, clinical attachment loss [38] and alveolar bone resorption [39], both of which indicate the cumulative history of periodontal disease, are associated with cognitive impairment. Moreover, systematic inflammatory reactions arising from periodontal disease are a hypothesized risk factor for Alzheimer’s disease [40,41]. Second, the association may depend on changes in food intake and nutrition status due to tooth loss. Poor nutritional status and nutrient deficiencies are reportedly associated with cognitive decline and Alzheimer’s disease [42,43]. Third, the association may arise from deteriorated masticatory performance resulting in reduced brain stimulation or cerebral blood flow [44,45]. Although the specifics of the process by which malocclusion causes delirium are not yet clear, we speculate that patients with the former potentially have cognitive dysfunction, including dementia, which is a risk factor of delirium (OR: 2.41) [30]. This study revealed that malocclusion on admission was significantly associated with the incidence of delirium in the elderly aged 65 years and older. Further studies are needed to clarify the detail association and mechanisms.

Notably, there are some study limitations. First, this was a retrospective study conducted in an acutely ill population from a single tertiary care center. Accordingly, the results may not be generalizable to less severely ill populations. In addition, we excluded 180 patients due to deficiencies in their sampling data. Of these, 100 were excluded due to deficiencies in their data concerning occlusal condition. These individuals tended to be younger and most of them were limb trauma patients; they were not examined by head-to-neck X-rays and oral assessments. Regarding the delirium analysis, we excluded 30 patients (9.4% in enrolled patients) because they were comatose (GCS < 9) and/or had severe dementia (CFS > 5) on admission, leading to less power in multivariate analysis. Second, we limited our focus to ADL loss and delirium as outcome variables; future studies may wish to explore other relevant outcomes. Third, a longer period of observation may be needed to assess the relationship between malocclusion and associated outcomes.

Despite these limitations, this is the first study to assess the relationship between malocclusion and ADL and the development of delirium for which poor oral health is a measure of short-term outcome in acutely ill patients. Moreover, poor oral health is frequently used as a bedside indicator of this clinical state. Furthermore, we believe it may be the intervenable factor in the ICU. Interventions for oral health can improve the clinical course by reducing malnutrition, secondary infections such as pneumonia, and delirium. Preventing tooth loss and encouraging denture wearing once teeth are lost may indirectly contribute to maintaining or improving ADL, mediated by recovery of swallowing function and nutritional status [29]. Further intervention studies may be needed to prove the association between oral frailty and not only short-term but also long-term ICU outcomes.

## 5. Conclusions

Malocclusion with molar teeth loss is associated with the loss of ADL loss and delirium in elderly critically ill patients. By recognizing this association, we hope to raise awareness of oral dysfunction prevention and strengthen medical and dental collaboration after hospitalization.

## Figures and Tables

**Table 1:** Baseline characteristics of the study population.

Variables	Total	Bilateral Occlusion	Malocclusion	*p* Value
N	320	227	93	
Age (years)	69 (57–79)	62 (48–74)	69 (64–77)	<0.01 *
Sex (male) (%)	179 (56)	128 (57)	51 (54)	0.62 ^#^
CFS on admission (%)				<0.01 ^##^
Scale 1	21 (7)	19 (8)	2 (2)	
Scale 2	101 (32)	83 (37)	19 (20)	
Scale 3	113 (35)	75 (33)	38 (41)	
Scale 4	57 (18)	32 (14)	25 (27)	
Scale 5	27 (8)	18 (8)	9 (10)	
APACHE II score	15 (11–21)	13 (10–18)	17 (13–24)	<0.01 *
Neurological disorders (%)	128 (40)	87 (39)	41 (43)	0.46 ^#^
Death in Hospital (%)	16 (5)	9 (4)	7 (7)	0.35 ^#^
ADL loss (%)	93 (29)	50 (22)	44 (46)	<0.01 ^#^
VFDs (mean ± SD, median)	21.2 ± 9.6, 27	22.6 ± 8.4, 27	18.1 ± 11.2, 24	0.50 *
N	290	209	81	
Delirium (%)	68 (23)	39 (19)	29 (36)	<0.01 ^#^

CFS, Clinical Frailty Scale; APACHE, Acute Physiology and Chronic Health Evaluation; ADL, Activities of Daily Living; VFDs, Ventilator-Free Days; SD, standard deviation. *: The Mann-Whitney U test was used. ^#^: The Fisher exact test was used. ^##^: Chi-squared test was used. Data are presented as number (percentage), or median (interquartile range).

**Table 2:** Baseline characteristics of the study population 65 years and older.

Variables	Total	Bilateral Occlusion	Malocclusion	*p* Value
N	171	103	68	
Age (years)	75 (69–82)	76 (69–82)	74.5 (69–79)	0.20 *
Sex (male) (%)	83 (49)	50 (49)	33 (49)	1.00 ^#^
CFS on admission (%)				<0.01 ^##^
Scale 1	0	0	0	
Scale 2	29 (17)	22 (21)	7 (10)	
Scale 3	73 (43)	41 (40)	32 (47)	
Scale 4	45 (26)	24 (23)	21 (31)	
Scale 5	24 (14)	16 (16)	8 (12)	
APACHE II score	16 (13–22)	15 (12–19)	19 (13–24)	<0.01 *
Neurological disorders (%)	74 (43)	49 (48)	25 (37)	0.16 ^#^
Death in Hospital (%)	10 (6)	4 (4)	4 (6)	0.42 ^#^
ADL loss (%)	58 (34)	26 (25)	32 (47)	<0.01 ^#^
N	153	93	60	
Delirium (%)	49 (32)	29 (31)	20 (33)	0.30 ^#^

CFS, Clinical Frailty Scale; APACHE, Acute Physiology and Chronic Health Evaluation; ADL, Activities of Daily Living; SD, standard deviation. *: The Mann-Whitney U test was used. ^#^: The Fisher exact test was used. ^##^: Chi-squared test was used. Data are presented as number (percentage), or median (interquartile range).

**Table 3:** Univariate and multivariate analyses for ADL loss of the study population.

	Univariate			Multivariate		
Variables	Crude OR	95% CI	*p* Value	Adjusted OR	95% CI	*p* Value
Age	1.03	1.00–1.04	<0.01	1.00	0.98–1.02	0.94
Sex (male)	1.03	0.63–1.67	0.89	1.30	0.74–2.31	0.35
CFS on admission	2.19	1.48–5.77	<0.01	1.94	1.40–2.71	<0.01
APACHE II score	1.12	1.04–2.71	<0.01	1.09	1.05–1.14	<0.01
Neurological disorders	2.03	1.24–3.30	<0.01	2.55	1.44–4.57	<0.01
Malocclusion	3.02	1.81–5.03	<0.01	2.03	1.13–3.64	0.02

CFS, Clinical Frailty Scale; APACHE, Acute Physiology and Chronic Health Evaluation; ADL, Activities of Daily Living; OR, odds ratio; CI, confidence interval; reference = bilateral occlusion.

**Table 4:** Univariate and multivariate analyses for ADL loss of the study population 65 years and older.

	Univariate			Multivariate		
Variables	Crude OR	95% CI	*p* Value	Adjusted OR	95% CI	*p* Value
Age	1.09	1.04–1.14	<0.01	1.08	1.02–1.15	<0.01
Sex (male)	0.89	0.47–1.67	0.71	1.11	0.51–2.40	0.80
CFS on admission	2.37	1.60–3.50	<0.01	2.20	1.38–3.49	<0.01
APACHE II score	1.09	1.03–1.14	<0.01	1.06	1.00–1.12	0.04
Neurological disorders	1.87	0.99–3.55	0.06	2.86	1.32–6.23	<0.01
Malocclusion	2.63	1.37–5.05	<0.01	3.25	1.44–7.32	<0.01

CFS, Clinical Frailty Scale; APACHE, Acute Physiology and Chronic Health Evaluation; ADL, Activities of Daily Living; OR, odds ratio; CI, confidence interval; reference = bilateral occlusion.

**Table 5:** Univariate and Multivariate analysis for Delirium of the study population.

	Univariate			Multivariate		
Variables	Crude OR	95% CI	*p* Value	Adjusted OR	95% CI	*p* Value
Age	1.03	1.01–1.05	<0.01	1.01	0.99–1.03	0.37
Sex (male)	0.89	0.56–1.43	0.64	1.04	0.61–1.76	0.88
CFS on admission	1.78	1.39–2.26	<0.01	1.40	1.05–1.95	0.02
APACHE II score	1.11	1.07–1.15	<0.01	1.09	1.05–1.13	<0.01
Neurological disorders	1.82	1.13–2.94	<0.01	1.97	1.16–3.36	0.01
Malocclusion	2.14	1.29–3.53	<0.01	1.33	0.76–2.34	0.32

CFS, Clinical Frailty Scale; APACHE, Acute Physiology and Chronic Health Evaluation; ADL, Activities of Daily Living; OR, odds ratio; CI, confidence interval; reference = bilateral occlusion.

**Table 6:** Univariate and Multivariate analysis for Delirium of the study population 65 years and older.

	Univariate			Multivariate		
Variables	Crude OR	95% CI	*p* Value	Adjusted OR	95% CI	*p* Value
Age	1.10	1.05–1.16	<0.01	1.13	1.06–1.20	<0.01
Sex (male)	0.89	0.48–1.67	0.72	1.18	0.55–2.54	0.68
CFS on admission	1.32	0.94–1.87	0.11	1.01	0.65–1.58	0.97
APACHE II score	1.06	1.01–1.10	0.01	1.07	1.01–1.13	0.02
Neurological disorders	4.03	2.07–7.84	<0.01	5.82	2.63–12.90	<0.01
Malocclusion	1.92	1.01–3.64	0.05	2.61	1.14–5.95	0.02

CFS, Clinical Frailty Scale; APACHE, Acute Physiology and Chronic Health Evaluation; ADL, Activities of Daily Living; OR, odds ratio; CI, confidence interval; reference = bilateral occlusion.

## Data Availability

The datasets generated and/or analyzed during the current study are available from the corresponding author on reasonable request.

## Appendix A

**Table A1:** Clinical Frailty Scale.

**1. Very Fit—**People who are robust, active, energetic, and motivated. People in this group exercise regularly and are among the fittest for their age.
**2. Well—**These people have no active disease symptoms but are less fit compared to those in category 1. They occasionally exercise or engage in activity, e.g., seasonally.
**3. Managing well—**People whose medical problems are well controlled; however, they are not regularly active beyond routine walking.
**4. Vulnerable—**While these people are not dependent on others for daily help, their symptoms often limit their activities. A common complaint is feeling “slowed down” and/or being tired during the day.
**5. Mildly frail—**These people often have more apparent slowing and need help with high order instrumental ADLs (finances, transportation, heavy housework, medications). Mild frailty progressively impairs the ability to shop, walk outside alone, prepare meals, and do housework.
**6. Moderately frail—**These people need help with all outside activities and with keeping house. They often have problems with stairs, need help with bathing, and might need minimal assistance (cueing, standby) with dressing.
**7. Severely frail—**These people are completely dependent on others for their personal care impairment. Even so, they appear to be stable and not at high risk of dying (within 6 months).
**8. Very Severely frail—**These people are completely dependent on others for their personal care as they are approaching the end of life. Typically, they could not even recover from a minor illness.
**9. Terminally ill—**These people are approaching the end of life. This category applies to people with a life expectancy <6 months who are not otherwise obviously frail.

## Appendix B

**Table A2:** Subgroup analysis by stratifying the level of CFS at admission to observe the difference of CFS in each level of ADL.

	Bilateral			Malocclusion			
CFS on Admission	*n*	CFS at Discharge (Difference of CFS)	Event (%)	*n*	CFS at Discharge (CFS Different)	Event (%)	*p* Value
1							0.34
	21	1 (0)	0	2	1 (0)	0	
		2 (1)	7 (33)		2 (1)	0	
		3 (2)	8 (38)		3 (2)	1 (50)	
		4 (3)	3 (14)		4 (3)	0	
		5 (4)	1 (5)		5 (4)	1 (50)	
		6–9 (5–8)	2 (10)		6–9 (5–8)	0	
2							0.09
	83	2 (0)	16 (19)	19	2 (0)	1 (5)	
		3 (1)	34 (41)		3 (1)	4 (21)	
		4 (2)	14 (17)		4 (2)	6 (32)	
		5 (3)	8 (10)		5 (3)	4 (21)	
		6–9 (4–7)	11 (13)		6–9 (4–6)	4 (21)	
3							0.02
	75	3 (0)	23 (31)	38	3 (0)	3 (8)	
		4 (1)	22 (29)		4 (1)	11 (29)	
		5 (2)	14 (19)		5 (2)	8 (21)	
		6–9 (3–6)	16 (21)		6–9 (3–5)	16 (42)	
4							0.14
	32	4 (0)	11 (34)	25	4 (0)	7 (25)	
		5 (1)	8 (25)		5 (1)	2 (8)	
		6–9 (2–5)	13 (41)		6–9 (2–4)	16 (64)	
5							0.20
	18	5 (0)	7 (39)	9	5 (0)	1 (11)	
		6–9 (1–4)	11 (61)		6–9 (1–3)	8 (89)	

CFS, Clinical Frailty Scale. *p* values were calculated by the Fisher exact test.

## Appendix C

**Table A3:** The quantifications of the interaction between the variables of the multivariate analysis.

	*p* for Interaction
ADL loss	
Age*Mal	<0.01
Sex (male)*Mal	0.07
APACHE*Mal	<0.01
Neuro*Mal	0.45
Delirium	
Age*Mal	<0.01
Sex (male)*Mal	0.438
APACHE*Mal	<0.01
Neuro*Mal	0.11

CFS, Clinical Frailty Scale; APACHE, Acute Physiology and Chronic Health Evaluation; ADL, Activities of Daily Living; Mal, Malocclusion.
